# Notes on Preparations for the Sick

**Published:** 1899-03

**Authors:** 


### Notes on Preparations for the Sick.

**Ichthalbin. Ichthyol Capsules. Desichthol.** — GUSTAV HERMANNI, Jr., London.—**Ichthalbin** is described as an "insipid powder for internal use, acting as an astringent and disinfectant on the bowels." It is presumably an ichthyol-albumin compound: it is practically tasteless, and should be of service in conditions where intestinal antiseptics are indicated. It does not contain any *soluble* albumin. **Ichthyol Capsules** are described as appetite promoting and nutrition increasing. **Desichthol** is an almost inodorous ichthyol, and is said to be analgesic and antiphlogistic in all forms of painful internal or external inflammation, but we have not been able to verify all these statements.

**Granular Effervescent Preparations: Bismuth, Pepsine, and Strychnia; Iron and Arsenic; Salicylate of Lithia.** — ALFRED BISHOP & SONS LTD., London.—Bishop's useful preparations are for the most part well known, and have been greatly in use for years. They often do good service in cases of anæmic and other forms of dyspepsia.

**Tabloids: Hypophosphites Compound; Zinc Oxide.**—BURROUGHS, WELLCOME & Co., London.—These new tabloids should be useful: of the former two strengths are issued, representing respectively half a drachm and one drachm of the compound syrup of hypophosphites. The rapid disintegration of the zinc oxide tabloid when dropped into water ensures its therapeutic activity. The convenience of the tabloid form must increase the utility of an often forgotten but valuable drug in the treatment of summer diarrhœa, gastralgia, epilepsy, chorea, and other results of nerve defects.

---

**Iron Somatose; Milk Somatose.**—FRIEDR. BAYER & Co., Elberfeld.—The first of these preparations is a light brown powder, soluble in water, and containing about 2 per cent. of iron. The dose is from 80 to 160 grains three times a day, and as the price is two shillings for one ounce it follows that this must be a very expensive method of giving iron in cases of anæmia. It does not therefore seem to be suitable to the ordinary case, but when other methods do not give satisfactory results this powder may be worthy of trial. The milk preparation contains 5 per cent. of tannic acid; it is therefore somewhat astringent, and more especially applicable to those patients who have a tendency to diarrhœa.

On the Somatose itself we commented in the *Journal* for September, 1895.

---

**Digestive Brown Bread; Diabetic Bread.**—J. S. NEWEY, Bristol.—Samples of these breads have been sent to us. They are excellent preparations, likely to be of use both in sickness and in health. The **Digestive Bread** is well named, inasmuch as it is made from malt flour mixed with wheat-meal flour, the malt being stewed for several hours until it attains a heat of 170 degrees. The bread is highly palatable, and it keeps moist and fresh for several days. Being made from whole meal, being well fermented and properly baked, and having the malt digestive intermixed, this bread should be highly nourishing and easily digested.

The **Diabetic Bread** is composed of aleuronat meal and cocoanut flour, mixed with eggs; the meal used in its preparation contains only a small percentage (5 to 7) of starch, and hence the product must be a valuable addition to the dietary of the diabetic patient, who is commonly very intolerant of the various kinds of gluten bread and cannot be induced to persevere with them. If we were diabetic we should feel no hardship in having to take bread like this; we cannot say that we prefer it to other kinds containing starch in quantity, but it is quite agreeable to the palate and retains its moisture well. We congratulate Mr. Newey on having accomplished—what appears to have been a difficulty hitherto—the manufacture in Bristol of an excellent bread for diabetics, which should obtain something more than a mere local reputation.

---

**Harzbach.**—BROWN BROTHERS & Co., Glasgow.—This comes to us accompanied with the details of an analysis made by Dr. E. J. Mills, F.R.S. Notwithstanding, or perhaps in consequence of, its manifold composition, it is a very palatable mineral water and it is well aërated. We mixed it with brandy and found in the compound a very pleasant reminder of Apollinaris under similar conditions, and this is giving it high praise.

---

**The "Copeman" Throat and Nasal Spray.**—S. Maw, Son, & Thompson, London.—This is a simple throat-spray without any indiarubber tubes, specially adapted for use in diphtheria. It is easy to use, and can be kept aseptic. The only objection to it that we can see is that the metal tube would be liable to be acted on by acids, but for many solutions it would be far more simple and cleanly than the ordinary form of throat-spray.

---

